# Genomic analysis of a nontoxigenic, invasive *Corynebacterium diphtheriae* strain from Brazil

**DOI:** 10.1590/0074-02760150250

**Published:** 2015-09

**Authors:** Fernando Encinas, Michel A Marin, Juliana N Ramos, Verônica V Vieira, Ana Luiza Mattos-Guaraldi, Ana Carolina P Vicente

**Affiliations:** 1 Fundação Oswaldo Cruz, Instituto Oswaldo Cruz, Laboratório de Genética Molecular de Microrganismos, Rio de Janeiro, RJ, Brasil; 2 Universidade do Estado do Rio de Janeiro, Faculdade de Medicina, Laboratório de Difteria e Corinebactérias de Importância Clínica, Rio de Janeiro, RJ, Brasil; 3 Fundação Oswaldo Cruz, Instituto Nacional de Controle de Qualidade em Saúde, Rio de Janeiro, RJ, Brasil

**Keywords:** Corynebacterium diphtheriae, ST-171, nontoxigenic

## Abstract

We report the complete genome sequence and analysis of an invasive *Corynebacterium diphtheriae* strain that caused endocarditis in Rio de Janeiro, Brazil. It was selected for sequencing on the basis of the current relevance of nontoxigenic strains for public health. The genomic information was explored in the context of diversity, plasticity and genetic relatedness with other contemporary strains.

*Corynebacterium diphtheriae* is the causative agent of diphtheria, a toxaemic disease that is controlled through immunisation programs. Infections caused by nontoxigenic strains of *C. diphtheriae* are not preventable by vaccination even though may cause severe invasive infections such as endocarditis and septic arthritis (Zasada et al. 2010, Peixoto et al. 2014). Outbreaks appear to be followed by periods of increasing genomic diversity (Mokrousov 2009) therefore, there is a need to explore the genomic information of these strains in terms of diversity and plasticity related to potential virulence determinants. In such a context, we describe the sequencing, annotation and analysis process of HC07, a nontoxigenic, invasive *C. diphtheriae* strain from Rio de Janeiro (RJ), Brazil.

HC07 was isolated in 2013 from the blood of a patient with endocarditis and belongs to biotype gravis. Identification and biotype determination were performed by conventional microbiological methods and the API Coryne System v.3.0 (bioMérieux, France) with the apiweb^TM^ web decoding system. HC07 was characterised as nontoxigenic by the modified Elek test at the Centers for Disease Control and Prevention (USA) and the Vero cell cytotoxicity assay. The multilocus sequence typing (MLST) scheme using seven housekeeping genes (*atp*A, *dna*E, *dna*K, *fus*A, *leu*A, *odh*A and *rpo*B) assigned the profile 2-2-36-19-3-3-6 that corresponds to ST-171. The HC07 genome was sequenced using a Nextera paired-end library in an Illumina HiSeq 2500 sequencer (Oswaldo Cruz Foundation high-throughput sequencing platform). Assemblies were generated with the A5-miseq pipeline v.20140604 (arxiv.org/abs/1401.5130) followed by a gene prediction and annotation process using the National Center for Biotechnology Information Prokaryotic Genomes Automatic Annotation Pipeline (Angiuoli et al. 2008). The shotgun project has been deposited at DDBJ/EMBL/GenBank under the accession JRUZ00000000 and version JRUZ00000000.1. The sequencing process rendered 16,360.560 reads (100 bp mean length) representing a 620x coverage, assembled into 70 contigs. Basic genomic features are as follows: total size, 2,491.635 bp, GC content, 53.53%, number of genes, 2,333, rRNAs, 6 (5S, 16S, 23S), and tRNAs, 51.

Phylogenetic reconstruction considering concatenated MLST genes (18,218 bp) showed that HC07 (gravis/isolated in 2013/ST-171) clusters with HC03 (mitis/isolated in 2000/ST-171) and HC04 (mitis/isolated in 2003/ST-128) (data not shown), these three strains are nontoxigenic and were isolated in RJ from patients with endocarditis. Moreover, a whole-genome based phylogeny also showed their close relationship (Fig. 1) supporting thus a previous report that biovar classification does not correlate with *C. diphtheriae* phylogeny (Sangal et al. 2014). All genomes were aligned using Progressive Mauve (Darling et al. 2010) and a neighbour-joining phylogenetic tree was constructed in SeaView (Gouy et al. 2010) on 16 complete *C. diphtheriae* genomes. Potential virulence factors in HC07 were mined using the Virulence Factors Database (mgc.ac.cn/VFs/). Two gene clusters were found: SpaA (NG01_07265-NG01_07280) and SpaH (NG01_09430; NG01_110470-NG01_11485) that encode a complete set of pilus proteins and their respective cognate sortases. Prophage and genomic island (GI) predictions were performed using the Phispy algorithm (Akhter et al. 2012) and the IslandViewer web server (pathogenomics.sfu.ca/islandviewer). HC07 harbours a putative prophage (NG01_04220-NG01_04530) that neither corresponds to the corynephage which usually carries the diphtheric toxin gene, nor to the one identified in HC03. Furthermore, 24 GIs were predicted and their genomic content is mostly represented by hypothetical proteins. Together, these results represent an overview of the diversity and plasticity of a nontoxigenic *C. diphtheriae* genome in terms of accessory gene content (Fig. 2).

**Fig. 1:**
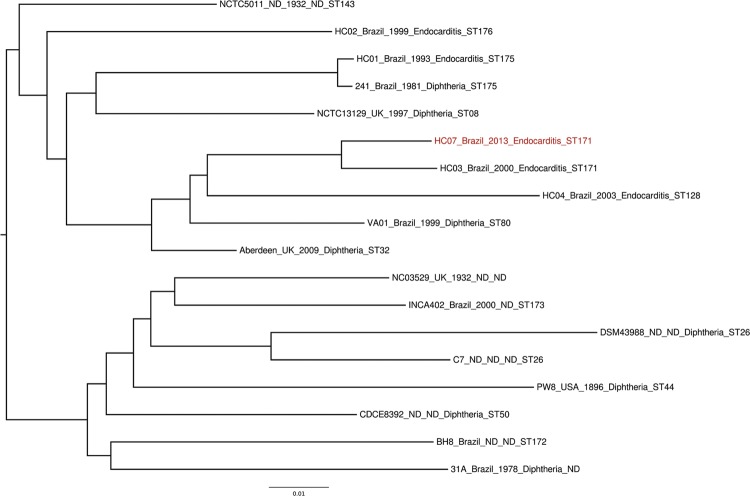
: phylogenomic tree including HC07 and other toxigenic/nontoxigenic *Corynebacterium diphtheriae*. Each genome is labelled by isolate name, country, year, disease and sequence type. ND: not determined.

**Fig. 2:**
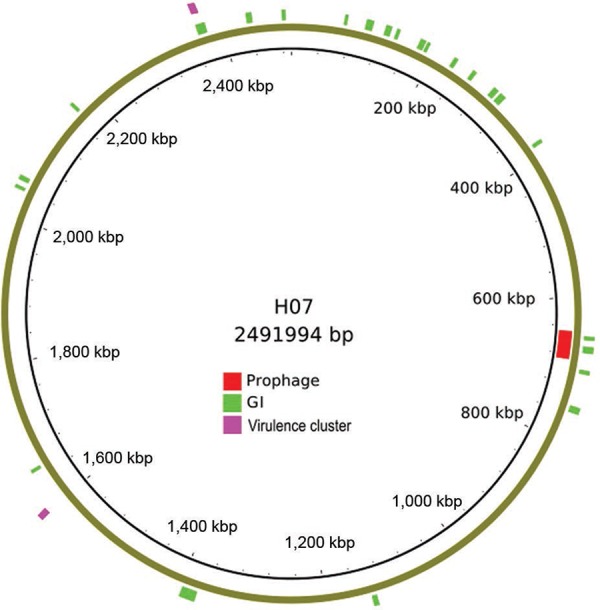
: circular representation of HC07 genome. Prophage, genomic islands (GIs) and virulence clusters are showed in red, green and purple, respectively.

Genomic analyses, including phylogenetic reconstructions, showed that *C. diphtheriae* HC07 clusters with other nontoxigenic endocarditis causative strains isolated in the same region more than a decade apart. This suggests the existence and persistence of a lineage that has been evolving and recurring in RJ. Particularly, HC07 harbours a set of potential virulence factors, a prophage and various GIs that characterise its diversity and plasticity that should be explored deeply in order to understand *C. diphtheriae *evolution.
